# N-Terminal Pro-B-Type Natriuretic Peptide as a Biomarker of Bronchopulmonary Dysplasia or Death in Preterm Infants: A Retrospective Cohort Analysis

**DOI:** 10.3389/fped.2019.00166

**Published:** 2019-05-07

**Authors:** Lin Zhou, Xiaowen Xiang, Li Wang, Xuting Chen, Jianxing Zhu, Hongping Xia

**Affiliations:** Department of Neonatology, Xinhua Hospital, Shanghai Jiao Tong University School of Medicine, Shanghai, China

**Keywords:** bronchopulmonary dysplasia, death, N-terminal pro-B-type natriuretic peptide, biomarker, preterm infant

## Abstract

**Objectives:** To investigate the association between serum N-terminal pro-B-type natriuretic peptide (NT-proBNP) level on the first day of life and a composite outcome of bronchopulmonary dysplasia (BPD) or death in a cohort of infants born before 32 weeks of gestation.

**Methods:** We retrospectively identified infants born before 32 weeks of gestation who had serum NT-proBNP levels measured when they were admitted to the Neonatal Intensive Care Unit shortly after birth. The outcome of BPD or death was assessed at 36 weeks of postmenstrual age. The association of serum NT-proBNP levels with BPD or death was evaluated. Receiver operator characteristic (ROC) curve analysis was used to evaluate the predictive performance of serum NT-proBNP levels.

**Results:** A 100 and 47 preterm infants had serum NT-proBNP levels measured on the first day of life. Serum NT-proBNP level was significantly higher in preterm infants who developed moderate/severe BPD or died [3,855 (2,567–6,369) vs. 1,259 (950–2,035) in control infants, *P* < 0.001]. On binary regression analysis, a high natural logarithm of serum NT-proBNP levels was associated with increased risk of moderate/severe BPD or death adjusted for gestational age, birth weight, birth weight z-score, and Apgar scores at 1 and 5 min (odds ratio [OR] = 5.195, 95% confidence interval [CI] 2.667–10.117, *P* < 0.001). ROC analysis identified a NT-proBNP level of 2002.5 pg/mL to have 87.5% sensitivity and 74.7% specificity for predicting moderate/severe BPD or death. The area under the curve (AUC) was 0.853 (95% CI 0.792–0.914).

**Conclusion:** Serum NT-proBNP level measured on the first day of life is a promising biomarker for predicting the development of moderate/severe BPD or death in preterm infants. Our findings warrant a larger prospective study to incorporate measurement of NT-proBNP in prognosticating outcomes in very preterm infants.

## Introduction

Bronchopulmonary dysplasia (BPD) is one of the most severe complications in preterm infants. Infants with BPD have higher rates of cognitive, educational, and behavioral impairments, and also have reduced lung function throughout childhood and into early life than would normally be expected (1). There is great interest in identifying a widely available biomarker with a strong prognostic capability that would allow for risk stratification in order to enable development of preventative strategies and early treatment for BPD.

B-type natriuretic peptide (BNP) is a hormone secreted from ventricular cardiomyocytes in response to volume or pressure overload and is involved in the regulation of extracellular fluid volume and blood pressure (2). N-terminal pro-BNP (NT-proBNP), the inactive by-product cleaved from proBNP, is more stable in serum sample and has a longer half-life in circulation. A pilot study including 34 infants born with a gestational age of < 34 weeks reported that higher serum NT-proBNP levels measured at 4 weeks of age in preterm infants was associated with an increased risk of BPD (3). Another cohort study included 60 infants born before 32 gestational weeks and showed that BNP was associated with BPD at the time of diagnosis (4). However, it is unknown whether NT-proBNP in early life is associated with BPD or death. We hypothesized that serum pro-BNP levels could identify preterm infants that have a high risk of BPD or death as early as the first day of life.

## Methods

### Data Collection

This retrospective cohort study was conducted in the Neonatal Intensive Care Unit at Xinhua Hospital Shanghai Jiao Tong University School of Medicine. Since 2015, most of the preterm infants had serum NT-proBNP level measurement, as it was included in the electrolytes, liver function, and renal function panels without requiring an extra blood sample. A total of 185 infants born before 32 weeks of gestation were hospitalized between January 2015 and December 2018 after excluding infants with any genetic disorder, congenital anomalies including complex congenital heart disease, or who died on the first day of life. Among them, 147 preterm infants with serum NT-proBNP measured on the first day of life were enrolled in our study. There was no difference in gestational age (GA) and birth weight (BW) between the participating and non-participating preterm infants [GA: 29^+2^ (28^+1^ to 31) vs. 29^+6^ (28^+4^ to 31) weeks, *P* = 0.448; BW: 1,214 ± 304 vs. 1,304 ± 323 grams, *P* = 0.080].

Data were collected from the infants' medical records and included gestational age, birth weight, birth weight z-score, gender, multiple birth, mode of delivery, maternal gestational hypertension (GH), maternal gestational diabetes mellitus (GDM), Apgar scores at 1 and 5 min, type of respiratory support (treatment with mechanical ventilation [MV] or with nasal continuous positive airway pressure [nCPAP]), sepsis, necrotizing enterocolitis (NEC), intraventricular hemorrhage (IVH) grades III–IV, and patent ductus arteriosus (PDA). The outcome was development of BPD or death. Birth weight z-scores were calculated by the methods of Fenton (5). According to the consensus definition by the National Institute of Child Health and Human Development (NICHD), BPD severity was graded as no, mild, moderate, or severe (defined as the need for oxygen supplementation or respiratory support at 36 weeks of postmenstrual age) (6).

### Statistical Analyses

Data analysis was performed with SPSS 21 (SPSS, Inc., Chicago, IL). Values are presented as numbers and percentages, mean ± standard deviation, or median and interquartile range (IQR, 25th and 75th percentile). Natural logarithm transformation was applied to the serum NT-proBNP levels to improve normality. A comparison of the characteristics in the infants with moderate/severe BPD or death and no/mild BPD was performed with independent samples *t*-test, Mann-Whitney *U*, or Fisher's exact test, as appropriate. The correlation between gestational age, birth weight and serum NT-proBNP levels was evaluated using Spearman analysis. One-way analysis of variance (ANOVA) compared the natural logarithm of serum NT-proBNP levels with gender, multiple birth, mode of delivery, maternal complications, and outcomes. Binary regression analysis was used to examine the association between BPD and gestational age, birth weight, birth weight z-scores, Apgar scores at 1 and 5 min, and the natural logarithm of the serum NT-proBNP levels. Receiver operating characteristic (ROC) analysis was performed to determine the serum NT-proBNP level that provided the best combination of sensitivity and specificity for predicting BPD or death. *P*-values of < 0.05 were considered to be statistically significant.

## Results

**Figure 1:**
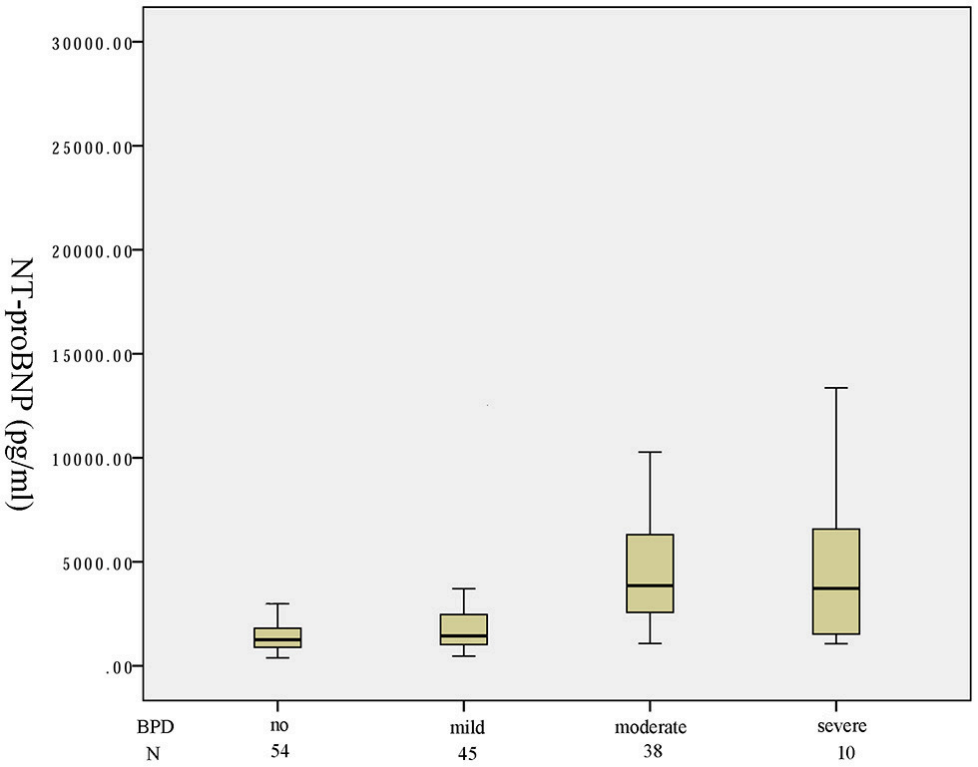
Serum NT-proBNP levels in four different groups of BPD severity.

One hundred and forty-seven preterm infants with a gestational age of < 32 weeks were identified in the study period. Fifty-four infants were not on any supplemental oxygen at 28 days of life and were characterized as no BPD. According to the NICHD classification of severity of BPD, 45, 38, 3 infants developed mild, moderate, severe BPD, respectively. Seven infants died before 36 weeks of postmenstrual age. A box plot of the median (IQR) serum NT-proBNP level in the four categories of no/mild/moderate/severe or death is shown in Figure 1. There was an increasing trend of serum NT-proBNP level with the severity of BPD. Serum NT-proBNP level was higher in infants who developed severe BPD or died and moderate BPD compared to mild BPD [4,102 (2,726–1,1665) vs. 1,438 (1,025–2,467), *P* = 0.005; 3,855 (2,603–6,182) vs. 1,438 (1,025–2,467), *P* < 0.001, respectively]. Serum NT-proBNP level was higher in infants who developed mild BPD compared to no BPD [1,438 (1,025–2,467) vs. 1,254 (894–1,763), *P* = 0.048]. There was no significant difference in the serum NT-proBNP level between those with severe BPD or death and moderate BPD (*P* = 0.755).

Table 1 shows a comparison of the clinical characteristics between the no/mild BPD group and the moderate/severe BPD or death group. There were no differences between the two groups for gender, multiple birth, mode of delivery, GH and GDM, respiratory support requirement, sepsis, and IVH grades III–IV. Infants with moderate/severe BPD or death were significantly more immature. The birth weight and birth weight z-scores in infants who developed moderate/severe BPD or died was significantly lower than that in infants with no/mild BPD. Infants who developed moderate/severe BPD or died had more unfavorable baseline characteristics (such as lower Apgar scores at 1 and 5 min) compared to infants with no/mild BPD. Infants who developed moderate/severe BPD or died also had higher morbidity of NEC and PDA. Serum NT-proBNP level was higher in infants who developed moderate/severe BPD or died compared to infants with no/mild BPD [3,855 (2,567–6,369) vs. 1,259 (950–2,035), *P* < 0.001].

**Table 1:** Clinical characteristics of the groups with and without BPD.

**Characteristics**	**All patients (*n* = 147)**	**No/mild BPD (*n* = 99)**	**Moderate/severe BPD or death (*n* = 48)**	***P*-value**
GA weeks, median (IQR)	29^+6^(28^+4^ to 31)	30 (28^+6^ to 31)	29^+1^(27^+2^ to 30^+6^)	0.013^*^
BW g, mean ± SD	1,304 ± 323	1,398 ± 304	1,110 ± 273	0.000^*^
BW Z-score, mean ± SD	−0.09 (−0.37 to 0.59)	0.28 (−0.23 to 0.6)	−0.17 (−0.59 to 0.45)	0.014^*^
Males, *n* (%)	78 (53)	55 (56)	23 (48)	0.384
Singleton, *n* (%)	93 (63)	66 (67)	27 (56)	0.219
Cesarean delivery, *n* (%)	94 (64)	61 (62)	33 (69)	0.398
Maternal GH, *n* (%)	33 (22)	18 (18)	15 (31)	0.075
Maternal GDM, *n* (%)	32 (22)	23 (23)	9 (19)	0.537
Apgar at 1 min, median (IQR)	8 (7–9)	9 (8–10)	7 (6–9)	0.000^*^
Apgar at 5 min, median (IQR)	10 (9–10)	10 (9–10)	9 (8–10)	0.002^*^
Surfactant, *n* (%)	128 (87)	86 (87)	42 (88)	0.915
MV, *n* (%)	122 (83)	80 (81)	42 (88)	0.311
nCPAP, *n* (%)	125 (85)	87 (88)	38 (79)	0.165
Sepsis, *n* (%)	10 (7)	5 (5)	5 (10)	0.226
NEC, *n* (%)	6 (4)	1 (1)	5 (10)	0.014^*^
IVH (III-IV), *n* (%)	9 (6)	3 (3)	6 (13)	0.059
PDA, *n* (%)	74 (50)	43 (43)	34 (65)	0.016^*^
NT-proBNP pg/ml, median (IQR)	1,883 (1,104 to 3,763)	1,259 (950 to 2,035)	3,855 (2,567 to 6,369)	0.000^*^
LnNT-proBNP, mean ± SD	7.45 ± 0.89	7.35 ± 0.76	8.38 ± 0.75	0.000^*^

*GA, gestational age; BW, birth weight; GH, gestational hypertension; GDM, gestational diabetes mellitus; MV, mechanical ventilation; nCPAP, nasal continuous positive airway pressure; NEC, necrotizing enterocolitis; IVH, intraventricular hemorrhage; PDA, patent ductus arteriosus; LnNT-proBNP, natural logarithm of serum NT-proBNP*.

* *indicates statistical significance*.

**Figure 2:**
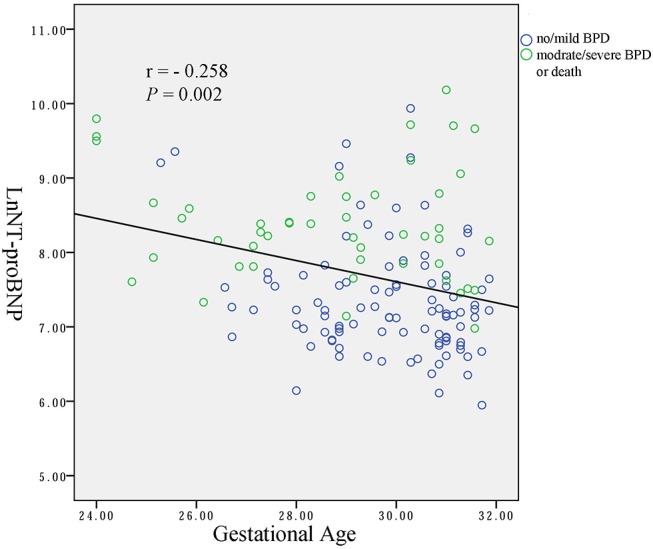
Correlation between natural logarithms of serum NT-proBNP level and gestational age.

**Figure 3:**
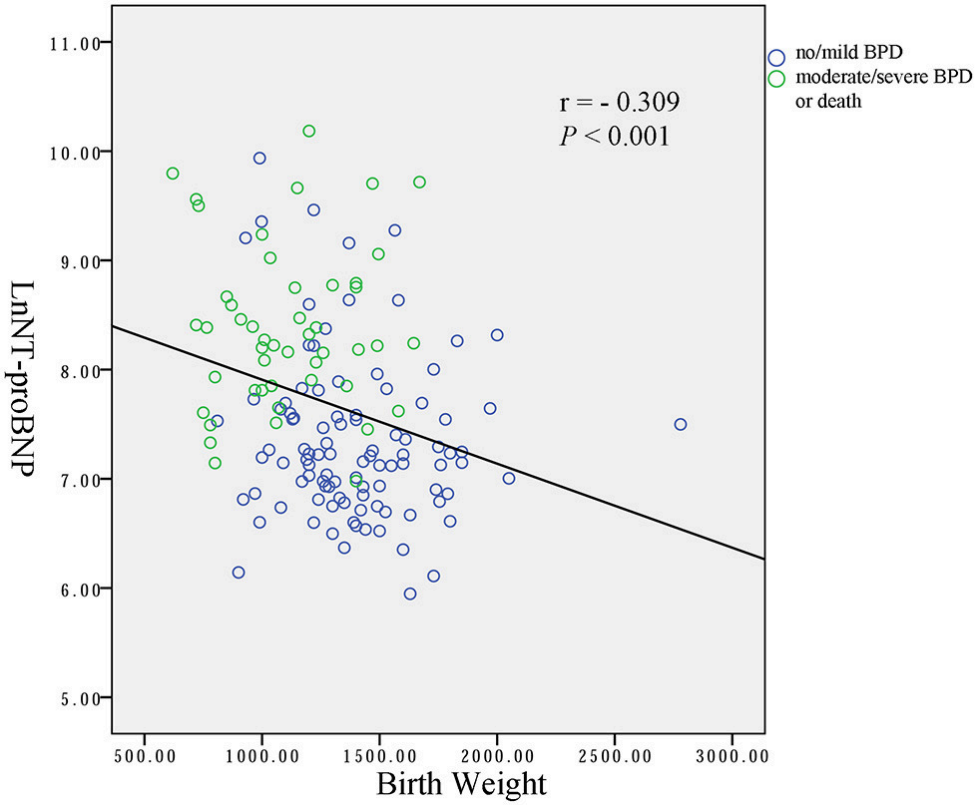
Correlation between natural logarithms of serum NT-proBNP level and birth weight.

Spearman analysis showed a negative correlation between the natural logarithms of serum NT-proBNP level and gestational age (*r* = −0.258, *P* = 0.002; Figure 2), and birth weight (*r* = −0.309, *P* < 0.001; Figure 3). We performed ANOVA to analyze the effect of clinical characteristics on serum NT-proBNP level. The association between the serum NT-proBNP level and gender, multiple birth, mode of delivery, maternal complications and morbidity of sepsis, NEC, IVH (III-IV), PDA, and BPD were examined (Table 2). It confirmed that serum NT-proBNP level was higher in infants who developed moderate/severe BPD or died compared to infants who with no/mild BPD. (*F* = 60.705, *P* < 0.001). It also revealed that serum NT-proBNP levels were higher in infants who developed IVH (III-IV) than those without IVH (III-IV) (*F* = 6.044, *P* = 0.015). There was approximately significant difference in serum NT-proBNP levels between infants who developed PDA and infants without PDA (*F* = 3.611, *P* = 0.059).

**Table 2:** Effects of clinical characteristics on serum NT-proBNP.

**Factors**	***n***	**NT-proBNP (pg/ml) median (IQR)**	**LnNT-proBNP mean ± SD**	***F*-value**	***P*-value**
Gender				3.583	0.060
Males	78	2,133 (1,079–4,315)	7.82 ± 1.00		
Females	69	1,806 (1,240–2,784)	7.54 ± 0.72		
Multiple birth				3.392	0.068
Singleton	93	1,749 (1,108–3,475)	7.58 ± 0.84		
Multiple birth	54	2,101 (1,117–5,334)	7.86 ± 0.94		
Mode of delivery				0.602	0.439
Cesarean delivery	94	1,980 (1,132–3,729)	7.73 ± 0.91		
Vaginal delivery	53	1,805 (1,020–3,763)	7.50 ± 0.85		
Maternal GH				0.597	0.441
GH	33	1,794 (1,067–3,643)	7.58 ± 0.87		
No GH	114	1,887 (1,237–3,779)	7.54 ± 0.90		
Maternal GDM				0.225	0.636
GDM	32	1,971 (1,357–2,957)	7.75 ± 0.91		
No GDM	115	1,863 (1,073–3,835)	7.67 ± 0.89		
Sepsis				0.160	0.690
Sepsis	10	2,233 (1,098–5,536)	7.80 ± 0.91		
No sepsis	137	1,883 (1,108–3,722)	7.68 ± 0.89		
NEC				1.631	0.204
NEC	6	3,649 (2,170–4,489)	7.14 ± 2.36		
No NEC	141	1,863 (1,101–3,722)	7.67 ± 0.89		
IVH (III–IV)				6.044	0.015^*^
IVH (III–IV)	9	4,382 (3,503–9,949)	8.38 ± 1.00		
No IVH (III–IV)	138	1,819 (1,103–3,710)	7.64 ± 0.87		
PDA				3.611	0.059
PDA	74	2,148 (1,073–4,382)	7.82 ± 0.96		
No PDA	73	1,749 (1,240–2,987)	7.55 ± 0.80		
BPD				60.705	0.000^*^
Moderate/severe BPD	48	3,855 (2,567–6,369)	8.38 ± 0.75		
No/mild BPD	99	1,259 (950–2,035)	7.35 ± 0.76		

*GH, gestational hypertension; GDM, gestational diabetes mellitus; NEC, necrotizing enterocolitis; IVH, intraventricular hemorrhage; PDA, patent ductus arteriosus*.

* *indicates statistical significance*.

**Figure 4:**
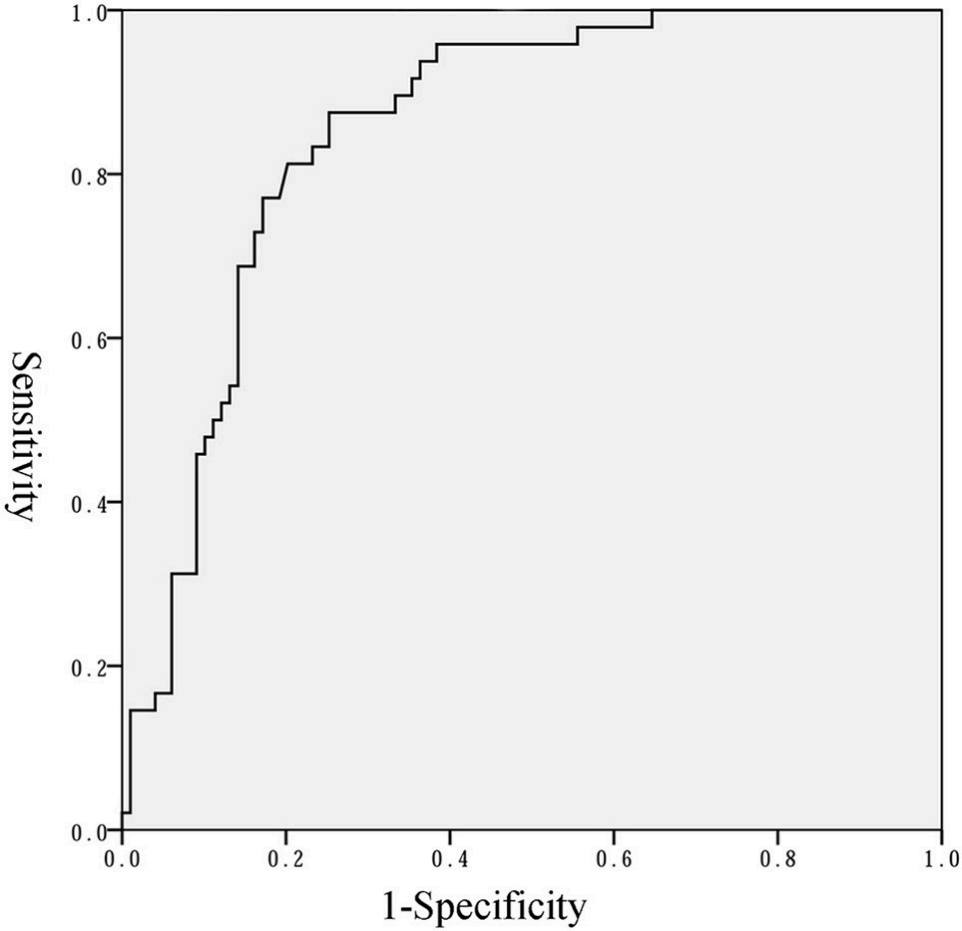
Receiver operator characteristics curve of NT-proBNP level to predict moderate/severe BPD or death.

Binary regression analysis showed that only the natural logarithm of the serum NT-proBNP level was associated with BPD or death adjusted for gestational age, birth weight, birth weight z-score and Apgar scores at 1 and 5 min (OR = 5.195, 95% CI = 2.667–10.117, *P* < 0.001). More statistical details are shown in Table 3. The ROC curve analysis shown in Figure 4 was used to identify a serum NT-proBNP cutoff value of 2002.5 pg/mL, which had the best combination of sensitivity (87.5%) and specificity (74.7%) for predicting moderate/severe BPD or death. The AUC was 0.853 (95% CI 0.792–0.914).

**Table 3:** Results of logistic regression for BPD.

**Parameter**	**OR**	**95% CI**	***P*-value**
GA	1.306	0.356–4.793	0.687
BW	0.995	0.987–1.004	0.260
BW z-score	0.878	0.076–10.086	0.917
Apgar scores at 1 min	0.763	0.460–1.264	0.293
Apgar scores at 5 min	1.512	0.675–3.38	0.315
Ln NT-proBNP	5.195	2.667–10.117	< 0.001

*OR, odds ratio; CI, Confidence Interval; GA, gestational age; BW, birth weight; LnNT-proBNP, natural logarithm of serum NT-proBNP*.

## Discussion

In this study, preterm infants with a gestational age of < 32 weeks at birth during the study period were enrolled. BPD or death was used as the outcome rather than BPD because death is a competing outcome with BPD (7). The results of this study showed that serum NT-proBNP level on the first day of life was significantly higher in infants who had moderate/severe BPD or died, than infants with no/mild BPD in very preterm infants. We also identified a serum NT-proBNP cutoff value of 2002.5 pg/mL to have the best combination of sensitivity (87.5%) and specificity (74.7%) for predicting moderate/severe BPD or death.

Elevated BNP or NT-proBNP levels have been reported in adult congestive heart failure (8), adult respiratory distress syndrome (9, 10) and adult pulmonary hypertension (11). The NT-proBNP measurement is valuable in the screening, diagnosis, management, and follow-up of children with cardiac disease (12). Neonatal infants with persistent pulmonary hypertension of the newborn (PPHN) have higher serum NT-proBNP levels than the infants without PPHN (13). The transition from fetal to neonatal circulation, lung expansion, and an elevation of systemic vascular resistance causes an increase of pulmonary blood flow. These perinatal circulatory changes lead to an increase in ventricular volume and pressure load and stimulate the BNP synthesis and secretion in the left and right atria and right ventricle after birth (14). In newborn infants, the plasma BNP levels were highest on the first day of life and decreased through maturation to stable levels comparable to adult levels at 3 months of age. The rapid increase of plasma BNP levels after birth may act to alleviate the increased left ventricular load and to support the myocardial function of the neonate (15).

Steurer et al. (16) reported that high BNP levels were associated with poor clinical outcome in newborns with congenital diaphragmatic hernia (CDH), because lung hypoplasia and abnormal development of the pulmonary vascular bed in infants with CDH may impact the transition to postnatal circulation in CDH and cause pulmonary hypertension elevated serum BNP levels. The severity of pulmonary hypertension in infants with CDH is related to developmental alterations in the pulmonary vascular bed (17, 18).

Puddy et al. (19) reported that early measurement of BNP in the first few days of life is a useful method for predicting preterm infants who had hemodynamically significant PDA. Plasma BNP levels correlate with changes in mean pulmonary arterial pressure during the first week of life in preterm infants (20). The magnitude of shunting through PDA is a major determinant of serum Plasma BNP level in preterm infants with hemodynamically significant PDA (21). El-Khuffash et al. (22) reported that high NT-proBNP and troponin T levels in infants with PDA were associated with an increased risk of intraventricular hemorrhage (IVH) or death. NT-proBNP level may be associated with sympathomimetic activity and cardiovascular compromise preceding the evolution of the hemorrhage (22). They also found that combining NT-proBNP and troponin T with echocardiography evaluates of PDA at 48 h might facilitate the identification of those infants with a PDA, who are at greatest risk of poor neurodevelopmental outcome at 2 years of age (23). Similarly, in our study, serum NT-proBNP levels were higher in infants who developed IVH (III–IV) than those without IVH (III–IV), and there was approximately significant difference in serum NT-proBNP levels between infants who developed PDA and infants without PDA.

Several studies have investigated the pathophysiological role of serum NT-proBNP and BNP levels in preterm infants with BPD. Sellmer et al. found that higher serum NT-proBNP levels in preterm infants at postnatal day 3 were associated with an increased risk of BPD or death in a study including of 183 infants born before 32 gestational weeks (24). Czernik et al. reported an association between urine NT-proBNP levels at the age of 7 days and BPD, defined as requiring supplemental oxygen at 4 weeks postnatal age, in 136 infants with a birth weight below 1,500 g (25). Serum NT-proBNP levels have been found to correlate with pulmonary hypertension (PH)-associated BPD (26). In a retrospective cohort study, Cuna et al. described an association between serum NT-proBNP levels and mortality in extremely low birth weight infants with PH-associated BPD (27). “New” BPD was characterized by interference in vascular and alveolar development, and dysregulation of pulmonary vascular development with fewer pulmonary vessels and structural remodeling (28). In preterm infants, excessive pulmonary pressure in an immature lung with ongoing maturation of alveolar and vascular structures may result in abnormal development of the pulmonary vessels (29). Pulmonary vessels may have increased responsiveness to oxygen, with mild hypoxia causing a marked elevation of pulmonary artery pressure (30). All of these hemodynamic changes may cause the development of BPD in preterm infants.

Our study was limited by its small sample population, retrospective nature and lack of longitudinal data. In recent years, the number of infants who are very preterm is increasing in China. However, the survival rate of extremely low birth weight infants (ELBWI) is much lower than that in developed countries. A multicenter study reported that ELBWI accounted for 15.1% of very low birth weight infants and the estimated overall mortality was 47.5% for ELBWI in China from May 2015 to April 2016 (31). Considering the poor long-term outcomes, some parents requested discontinuation of therapy for their infants who were very preterm. Preterm infants in our study had larger gestational age and birth weight than those with BPD in other studies. A prospective study involving a larger sample size is needed to incorporate measurements of NT-proBNP in prognosticating outcomes in infants who are very preterm.

## Conclusion

We found that the likelihood of BPD or death was associated with high serum NT-proBNP levels measured on the first day of life. If our data are confirmed in a larger prospective study, serum NT-proBNP estimation may be a promising biomarker of BPD or death in preterm infants.

## Ethics Statement

This study was exempt from informed consent standards due to the use of patients' biochemical and echographic data being obtained from past health questionnaire database. This study did not constitute a risk for the patients and was approved by the ethics committees of Xinhua Hospital.

## Author Contributions

HX conceived and designed the study. JZ and HX revised the manuscript. LZ, XX, LW, and XC collected the information. LZ and XX performed the data analysis and wrote the manuscript. LZ and XX contributed equally to this work. All authors reviewed the manuscript.

### Conflict of Interest Statement

The authors declare that the research was conducted in the absence of any commercial or financial relationships that could be construed as a potential conflict of interest.

## Abbreviations

NT-proBNP: N-terminal pro-B-type natriuretic peptide.

## References

[1] Doyle LW Anderson PJ. Long-term outcomes of bronchopulmonary dysplasia. Semin Fetal Neonatal Med. (2009) 14:391–5. 10.1016/j.siny.2009.08.004 19766550

[2] Levin ER Gardner DG Samson WK. Natriuretic peptides. N Engl J Med. (1998) 339:321–8. 10.1056/NEJM199807303390507 9682046

[3] Joseph L Nir A Hammerman C Goldberg S Ben Shalom E Picard E. N-terminal pro-B-type natriuretic peptide as a marker of bronchopulmonary dysplasia in premature infants. Am J Perinatol. (2010) 27:381–6. 10.1055/s-0029-1243312 20013606

[4] Kalra VK Aggarwal S Arora P Natarajan G. B-type natriuretic peptide levels in preterm neonates with bronchopulmonary dysplasia: a marker of severity? Pediatr Pulmonol. (2014) 49:1106–11. 10.1002/ppul.22942 24214578

[5] Fenton TR Kim JH. A systematic review and meta-analysis to revise the Fenton growth chart for preterm infants. BMC Pediatr. (2013) 13:59. 10.1186/1471-2431-13-59 23601190 PMC3637477

[6] Jobe AH Bancalari E. Bronchopulmonary dysplasia. Am J Respir Crit Care Med. (2001) 163:1723–9. 10.1164/ajrccm.163.7.2011060 11401896

[7] Andersen PK Geskus RB de Witte T Putter H. Competing risks in epidemiology: possibilities and pitfalls. Int J Epidemiol. (2012) 41:861–70. 10.1093/ije/dyr213 22253319 PMC3396320

[8] Gotze JP Kastrup J. Plasma pro-brain natriuretic peptides are strong biochemical markers in clinical cardiology. Scand J Clin Lab Invest Suppl. (2001) 234:47–51. 10.1080/clb.61.234.47.51 11713980

[9] Mitaka C Hirata Y Nagura T Tsunoda Y Itoh M Amaha K. Increased plasma concentrations of brain natriuretic peptide in patients with acute lung injury. J Crit Care. (1997) 12:66–71. 10.1016/S0883-9441(97)90003-4 9165414

[10] Maeder M Ammann P Rickli H Diethelm M. Elevation of B-type natriuretic peptide levels in acute respiratory distress syndrome. Swiss Med Wkly. (2003) 133:515–8. 14652800 10.4414/smw.2003.10367

[11] Nagaya N Nishikimi T Uematsu M Satoh T Kyotani S Sakamaki F. Plasma brain natriuretic peptide as a prognostic indicator in patients with primary pulmonary hypertension. Circulation. (2000) 102:865–70. 10.1161/01.cir.102.8.865 10952954

[12] Cantinotti M Walters HL Crocetti M Marotta M Murzi B Clerico A. BNP in children with congenital cardiac disease: is there now sufficient evidence for its routine use? Cardiol Young. (2015) 25:424–37. 10.1017/S1047951114002133 25601330

[13] Baptista MJ Correia-Pinto J Rocha G Guimaraes H Areias JC. Brain-type natriuretic peptide in the diagnosis and management of persistent pulmonary hypertension of the newborn. Pediatrics. (2005) 115:1111. 10.1542/peds.2004-2780 15805406

[14] Mir TS Laux R Hellwege HH Liedke B Heinze C von Buelow H. Plasma concentrations of aminoterminal pro atrial natriuretic peptide and aminoterminal pro brain natriuretic peptide in healthy neonates: marked and rapid increase after birth. Pediatrics. (2003) 112:896–9. 10.1542/peds.112.4.896 14523183

[15] Yoshibayashi M Kamiya T Saito Y Nakao K Nishioka K Temma S. Plasma brain natriuretic peptide concentrations in healthy children from birth to adolescence: marked and rapid increase after birth. Eur J Endocrinol. (1995) 133:207–9. 10.1530/eje.0.1330207 7655645

[16] Steurer MA Moon-Grady AJ Fineman JR Sun CE Lusk LA Wai KC. B-type natriuretic peptide: prognostic marker in congenital diaphragmatic hernia. Pediatr Res. (2014) 76:549–54. 10.1038/pr.2014.136 25188741 PMC4232979

[17] Vacanti JP Crone RK Murphy JD Smith SD Black PR Reid L. The pulmonary hemodynamic response to perioperative anesthesia in the treatment of high-risk infants with congenital diaphragmatic hernia. J Pediatr Surg. (1984) 19:672–9. 10.1016/S0022-3468(84)80351-6 6520671

[18] Geggel RL Murphy JD Langleben D Crone RK Vacanti JP Reid LM. Congenital diaphragmatic hernia: arterial structural changes and persistent pulmonary hypertension after surgical repair. J Pediatr. (1985) 107:457–64. 10.1016/S0022-3476(85)80534-5 4032138

[19] Puddy VF Amirmansour C Williams AF Singer DR. Plasma brain natriuretic peptide as a predictor of haemodynamically significant patent ductus arteriosus in preterm infants. Clin Sci. (2002) 103:75–7. 10.1042/cs1030075 12095406

[20] Ikemoto Y Nogi S Teraguchi M Kojima T Hirata Y Kobayashi Y Early changes in plasma brain and atrial natriuretic peptide in premature infants: correlation with pulmonary arterial pressure. Early Hum. Dev. (1996) 46:55–62. 10.1016/0378-3782(96)01741-0 8899354

[21] Holmstro6m H Hall C Thaulow E Plasma levels of natriuretic peptides and hemodynamic assessment of patent ductus arteriosus in preterm infants. Acta Paediatr. (2001) 90:184–91. 10.1080/080352501300049406 11236049

[22] El-Khuffash A Barry D Walsh K Davis PG Molloy EJ Biochemical markers may identify preterm infants with a patent ductus arteriosus at high risk of death or severe intraventricular haemorrhage. Arch Dis Child Fetal Neonatal Ed. (2008) 93:F407–12. 10.1136/adc.2007.133140 18285375

[23] El-Khuffash AF Slevin M McNamara PJ Molloy EJ Troponin T, N-terminal pro natriuretic peptide and a patent ductus arteriosus scoring system predict death before discharge or neurodevelopmental outcome at 2 years in preterm infants. Arch Dis Child Fetal Neonatal Ed. (2011) 96:F133–7. 10.1136/adc.2010.185967 21071684

[24] Sellmer A Hjortdal VE Bjerre JV Schmidt MR McNamara PJ Bech BH N-terminal pro-B type natriuretic peptide as a marker of bronchopulmonary dysplasia or death in very preterm neonates: a cohort study. PLoS ONE. (2015) 10:e0140079 10.1371/journal.pone.0140079 26452045 PMC4599729

[25] Czernik C Metze B Muller C Muller B Buhrer C. Urinary N-terminal B-type natriuretic peptide predicts severe retinopathy of prematurity. Pediatrics. (2011) 128:e545–9. 10.1542/peds.2011-0603 21824875

[26] Montgomery AM Bazzy-Asaad A Asnes JD Bizzarro MJ Ehrenkranz RA Weismann CG. Biochemical screening for pulmonary hypertension in preterm infants with bronchopulmonary dysplasia. Neonatology. (2016) 109:190–4. 10.1159/000442043 26780635

[27] Cuna A Kandasamy J Sims B. B-type natriuretic peptide and mortality in extremely low birth weight infants with pulmonary hypertension: a retrospective cohort analysis. BMC Pediatr. (2014) 14:68. 10.1186/1471-2431-14-68 24612708 PMC3975241

[28] Jobe AJ. The new BPD: an arrest of lung development. Pediatr Res. (1999) 46:641–3. 10.1203/00006450-199912000-00007 10590017

[29] Mata-Greenwood E Meyrick B Soifer SJ Fineman JR Black SM. Expression of VEGF and its receptors Flt-1 and Flk-1/KDR is altered in lambs with increased pulmonary blood flow and pulmonary hypertension. Am J Physiol Lung Cell Mol Physiol. (2003) 285:L222–31. 10.1152/ajplung.00388.2002 12665467

[30] Mourani PM Ivy DD Gao D Abman SH. Pulmonary vascular effects of inhaled nitric oxide and oxygen tension in bronchopulmonary dysplasia. Am J Respir Crit Care Med. (2004) 170:1006–13. 10.1164/rccm.200310-1483OC 15184202

[31] Reduction of Infection in Neonatal intensive care units using the Evidence-based Practice for Improving Quality (REIN-EPIQ) Study Group Outcomes of very low birth weight infants at discharge: a multicentered cross-sectional study of 25 tertiary neonatal intensive care units in China. Chin J Perinat Med. (2018) 21:394–400. 10.3760/cma.j.issn.1007-9408.2018.06.007

